# Assessment of Ammonia Adsorption Capacity on Activated Banana Peel Biochars

**DOI:** 10.3390/ma18143395

**Published:** 2025-07-20

**Authors:** Katarzyna Jedynak, Barbara Charmas

**Affiliations:** 1 Institute of Chemistry, Faculty of Exact and Natural Sciences, Jan Kochanowski University, Uniwersytecka Str. 7, 25-406 Kielce, Poland; katarzyna.jedynak@ujk.edu.pl; 2 Institute of Chemical Sciences, Faculty of Chemistry, Maria Curie-Sklodowska University, Maria Curie-Sklodowska Sq. 3, 20-031 Lublin, Poland

**Keywords:** activated biochar, banana peels, chemical activation, adsorption gas-phase pollutants, ammonia gas

## Abstract

This paper presents the assessment of the possibility of ammonia adsorption on biochars from banana peels, chemically activated with potassium hydroxide (KOH) at different temperatures. The obtained materials were characterized in detail using a number of analytical techniques, including nitrogen adsorption (BET), scanning electron microscopy (SEM), elemental analysis (CHNS), thermal analysis (TG, DTG, DTA), Fourier transform infrared spectroscopy (ATR-FTIR), Raman spectroscopy, Boehm titration method and biochar surface pH. They revealed a largely developed microporous structure and a large specific surface area, ranging from 1134 to 2332 m^2^ g^−1^. The adsorption tests against ammonia in the gas phase showed a large adsorption capacity of the materials, up to 5.94 mmol g^−1^ at 0 °C and 3.83 mmol g^−1^ at 20 °C. The adsorption properties of the obtained biochars were confirmed to be significantly influenced by the surface chemistry (presence of the acidic functional groups). The research results indicate that the waste-based biomass, such as banana peels, can be an ecological and economical raw material for the production of highly effective adsorbents, useful in the removal of ammonia and other toxic gases polluting the environment.

## 1. Introduction

The production of municipal solid waste is increasing from year to year due to living standards improvement, population growth and urbanization. Most waste is organic matter, and traditional methods of disposal include landfilling, incineration as well as composting. The cost of solid waste management is very large. Moreover, this type of organic waste injures human health and the environment, producing greenhouse gases and is the cause of numerous diseases [1]. An alternative is to use this type of matter to produce biochars. Biochar is a solid, carbon-rich material formed as a result of biomass decomposition under the limited oxygen access conditions, usually in the temperature range 500–800 °C [2,3,4]. The most common technology for biochar production is pyrolysis—a process carried out in various variants: fast, intermediate, or slow. Alternatively, such methods as gasification or hydrothermal carbonization are also applied [5,6,7].

The biochar production process is fast and economically viable, mainly due to the large value of the obtained final product. Additionally, biochar has some environmental and agricultural benefits. It can contribute to improving soil structure and fertility, support seed germination and stimulate plant growth. It also increases the soil resistance to diseases and its ability to retain water as well as helps to remove toxic substances. Moreover, biochar is used as an ecological fuel and an effective carbon store, supporting climate protection activities [8,9,10]. It is produced using both specially grown biomass and a wide range of waste materials, i.e., from the agricultural industry. Examples of such raw materials are manure, plant debris, pruned branches, various types of wood, sewage sludge and municipal waste [11,12,13], cotton stalks [14], rice husk [15], cones [16,17], corn cobs and corn stalk [18], sawdust [19,20,21], and many others.

The chemical composition and structure of the biomass from which the biochar is obtained play an important role in shaping the properties of the final product. Lignin, cellulose and hemicellulose contained in it—the compounds characterized by large resistance to large temperatures—are particularly important. Lignin, as the most durable of them, is characterized by a high content of polyaromatic structures and remains stable even above 300 °C. Its quantitative and qualitative contribution depends on the type of biomass, which affects the final properties of the produced biochar [22,23].

In order to obtain biochar with the desired properties, i.e., with appropriate morphological and structural parameters, the precursor used for its production should be characterized by a small ash content and, above all, a large carbon content [24,25]. Biochar-based adsorbents obtained from natural resources due to their properties: adjustable porosity, the presence of numerous functional groups, the profitability of production and the possibility of their regeneration, are commonly used for environmental remediation [26]. However, biochars obtained by direct pyrolysis at a high temperature from numerous biomass wastes under anaerobic conditions have small porosity, which is also reflected in the small specific surface area and results in insufficient adsorption properties. Therefore, it is important to develop porosity by activating biochar materials [27]. The activation process can be carried out using two basic methods: physical and chemical ones [28]. Physical activation usually takes place in two stages. The first stage is the carbonization of the carbon raw material in an inert gas atmosphere. Then, the material is subjected to a high temperature in the presence of an oxidizing gas, such as CO_2_, water vapor, air, or a mixture of these [29,30].

In the case of chemical activation there are applied two technological approaches. The first one involves impregnation of the carbon precursor with an appropriate chemical activator and then its thermal treatment, during which both carbonization and activation take place [31]. The second approach separates these steps: first, the material is carbonized in the temperature range of 300–600 °C, and then impregnated with a chemical agent and activated at a higher temperature, usually in the range of 700–1200 °C [32].

In the era of growing problems related to the environmental pollution, cheap and effective technologies enabling the reduction in harmful gas emissions are gaining particular importance. Pollutants present in the air and also spread indoors through ventilation systems can have a negative impact on people’s health, regardless of whether they are outdoors or in confined spaces [33]. Particularly noteworthy are polluting gases, including ammonia, which pose a risk of creating health problems. Ammonia (NH_3_) is a colorless, toxic gas with a characteristic, pungent odor, lighter than air. Even low concentrations of ammonia (with an acceptable exposure limit of 50 ppm) can lead to skin and eye irritation, while long-term exposure is associated with the risk of serious damage to the internal organs, such as lungs, kidneys, liver or brain, as well as metabolic disorders [1,33]. This gas, which is hazardous to living organisms and the atmospheric environment, primarily originates from agricultural activities, transportation, and numerous industrial processes [34]. Ammonia causes eutrophication of water bodies [35] as well as contributes to the formation of fog in the atmosphere [36]. Moreover, working with this corrosive gas poses a risk of danger, as it can lead to corrosion of the research equipment [37].

Banana peels are a type of waste with a great potential for the production of high value-added materials such as biochars [1,38]. They are characterized by a large content of organic matter, mainly cellulose, hemicellulose, lignin as well as phenolic compounds and pectins. These compounds, subjected to the process of pyrolysis or carbonization under the controlled conditions, undergo thermochemical degradation, leading to formation of biochar with a diverse porous structure and the presence of active surface groups, such as hydroxyl (–OH), carboxyl (–COOH) or carbonyl (C=O) ones. This type of waste is part of the solid waste generated during the consumption and processing of this fruit. They are a source of pollution, they do not provide any economic benefits, but only carry on the environmental pollution. This type of biomass is a problem in terms of utilization and storage [39].

The aim of this study was to obtain chemically activated biochars from banana peels. The influence of the type of biomass and the activation temperature on the key physicochemical properties of the obtained adsorbents and their ability to remove gaseous pollutants, with particular emphasis on ammonia, was analyzed. Effective reduction in ammonia concentrations in the atmosphere is an important issue from the point of view of both environmental protection and public health. In recent years, more and more attention has been paid to carbon materials with large adsorption capacity, which can be used in the processes of removing toxic gases from the air, including ammonia.

The paper makes important contribution to the current knowledge on low-cost and sustainable methods of obtaining adsorption materials that exhibit the adsorption potential against ammonia. For the first time, a comprehensive assessment of the adsorption capacity of ammonia on activated biochars obtained from banana peels, an organic waste that is widely available but rarely used in this context, was conducted. There were applied different activation conditions, which resulted in the materials characterized by optimized textural and surface properties. The influence of activation temperature on the efficiency of NH_3_ adsorption was investigated. The results proved that the banana peel biochar can compete with the conventional adsorbents, offering low cost and great efficiency while managing agricultural waste.

## 2. Materials and Methods

### 2.1. Materials

Banana peels collected from the household were used as a carbon precursor. All chemicals (KOH, HCl, NaOH) were purchased from POCH (Gliwice, Poland).

### 2.2. Biochar Preparation

**Figure 1:**
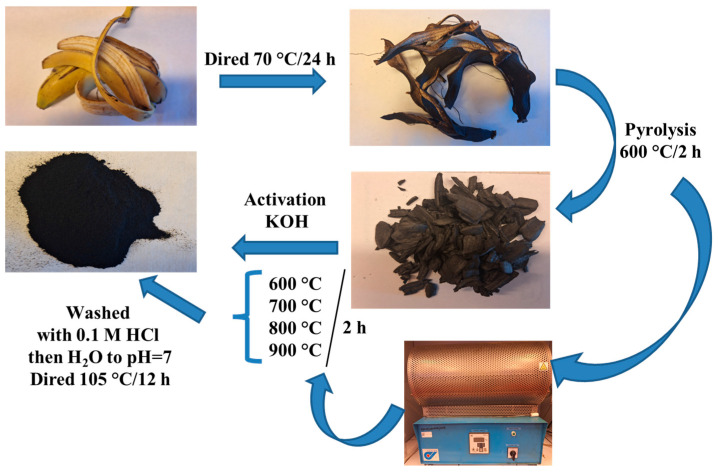
Scheme of the activated biochars preparation.

The banana peels were dried in a drying oven (BINDER GmbH, Tuttlingen, Germany) at 70 °C for 24 h. Then, the material was crushed into pieces and used for further research. Stage I: The pyrolysis was conducted in a nitrogen atmosphere (flow rate 20 dm^3^ h^−1^), from room temperature to 600 °C (rate of heating: 5 °C min^−1^) and maintained at this temperature 2 h (tube furnace by Czylok, Poland). The obtained biochar was designated B. Stage II: At the beginning, the biochar was ground with KOH at the weight ratio KOH:C 4:1. Then, the product was placed in quartz boats and the process of biochar activation was conducted at four different temperatures: 600, 700, 800 and 900 °C in atmosphere of nitrogen (flow rate 20 dm^3^ h^−1^, heating rate 5 °C min^−1^) for 2 h. After this stage, the materials were washed with 0.1 M HCl and then washed to neutral pH with water, after which they were dried at 105 °C for 12 h. The resulting activated biochars were designated: B–KOH–600; B–KOH–700; B–KOH–800; B–KOH–900. The scheme of the applied procedures is given in Figure 1.

### 2.3. Methods

#### 2.3.1. The Nitrogen Low-Temperature Adsorption/Desorption

The porous characteristics of the carbon materials were estimated based on the nitrogen adsorption-desorption isotherms at −196 °C, utilizing a volumetric adsorption analyzer (ASAP 2020, Micromeritics, Norcross, GA, USA). Before the analysis, all samples were degassed at 200 °C for 2 h to remove physically adsorbed species. Based on the obtained nitrogen adsorption data, the standard textural parameters: specific surface area (S_BET_), total pore volume (V_t_), and pore size distribution (PSD) were determined. The Brunauer–Emmett–Teller method was employed to calculate the specific surface area (S_BET_) in the relative pressure (p/p_0_) range of 0.05–0.20 [40]. The V_t_ was estimated from the adsorption data at a relative pressure of p/p_0_ = 0.99 [41]. The PSD functions were derived using the non-local density functional theory (NLDFT), which accounts for surface energy heterogeneity and structural corrugation of slit-like pores in materials using the SAIEUS software 3.0 (Micromeritics) [42,43].

#### 2.3.2. SEM/EDS Analysis

The biochar morphology was analyzed using a scanning electron microscope (DualBeam Quanta 3D FEG FEI (FEI Company, Hillsboro, NE, USA) with an accelerating voltage of 5 kV.

#### 2.3.3. CHNS Analysis

The elemental composition (CHNS) was determined using a Vario Micro Cube analyzer (Elemental, Langenselbold, Germany). Prior to analysis, all samples needed to be dried to a constant weight to ensure the accuracy of the results.

#### 2.3.4. ATR-FTIR

FTIR analysis was performed using a Perkin-Elmer Spectrum 400-FT-IR/FT-NIR spectrometer (Perkin-Elmer, Waltham, MA, USA) equipped with a single-bounce diamond ATR (attenuated total reflection) accessory. Spectra were obtained in the range of 4000–650 cm^−1^ by co-adding 500 scans at a spectral resolution of 4 cm^−1^ . Before the measurement, all samples were dried and finely ground using an agate mortar.

#### 2.3.5. Raman Spectroscopy

The structural ordering of the carbon framework was assessed by recording Raman spectra using a Raman Station 400 F spectrometer (Perkin Elmer, Waltham, MA, USA), equipped with a thermoelectrically cooled charge-coupled device (CCD) detector and a diode laser. Measurements were made at a wavelength of 785 nm with a laser power of 350 mW. Prior to the analysis, all samples were thoroughly dried. For each sample, five individual scans were collected with a scanning duration of 20 s per scan. The spectral resolution was maintained at 1 cm^−1^ .

#### 2.3.6. Boehm Method

The Boehm titration method [44,45,46] was utilized for the quantification of surface acidic and basic oxygen-containing functional groups. The biochar samples (0.2 g) were suspended in the 0.1 mol dm^−3^ sodium hydroxide solution (25 cm^3^) for acidic group determination, while the 0.05 mol dm^−3^ hydrochloric acid solution (25 cm^3^) was used to assess total basic groups. The suspensions were shaken at room temperature for 48 h, filtered, and 10 cm^3^ of the filtrate was titrated with 0.1 mol dm^−3^ HCl or 0.05 mol dm^−3^ NaOH to determine the acidic or basic groups, respectively. Titrations were performed using a TitroLine easy automatic titrator (SCHOTT Instruments, Mainz, Germany).

#### 2.3.7. Thermal Analysis

Thermal stability and the content of volatile and fixed carbon were determined using Derivatograph C (Paulik, Paulik & Erdey, MOM, Budapest, Hungary). The samples (~20 mg) were placed in a corundum crucible. Al_2_O_3_ was a reference material. The measurements were carried out in air or inert (N_2_) atmospheres in the temperature range from 20 to 1000 °C (heating rate 10 °C min^−1^). The content of less humified matter (volatile carbon, %VC) was determined from the thermogravimetric analysis (TGA) data in the N_2_ atmosphere in the temperature range 200–900 °C, assuming that moisture is desorbed up to 200 °C. The content of ashes (%A) was determined as an inorganic residue after the complete thermo-oxidation of the biochar in an O_2_ atmosphere at 1200 °C. The more humified organic matter content (fixed carbon, %FC) was determined as the difference of TG% _1200,O_2_,_ and TG% _900,N2_ [47]. The thermostable fraction (C_thermo_, poorly thermodegradable, ash-free, to dry mass) was determined as the content of stable matter (%FC) to the sum of volatile (%VC) and fixed substances (%FC) [48]. This parameter allows for assessing organic matter stability in biochars. For better characterization of carbon transformation, the TG data were corrected for moisture and ash content [49].

#### 2.3.8. Surface pH

The pH of the biochars was measured according to the procedure outlined by Nowicki et al. [50]. Approximately 0.4 g of the biochar was mixed with 20 cm^3^ of distilled water and shaken at 25 °C and 140 rpm for 24 h. Next, the pH of the resulting supernatants was determined.

#### 2.3.9. Chemisorption

The measurements of pulse chemisorption and temperature-programmed desorption (TPD) of ammonia were performed using an automated system (AutoChem II 2920, Micromeritics, Norcross, GA, USA). Before the measurements, all biochars were degassed (200 °C, 2 h, ASAP 2020, Micromeritics, Norcross, GA, USA). In the initial step, 50 mg of biochar was stabilized in a quartz reactor under a helium flow at 250 °C (heating rate: 20 °C min^−1^ for 40 min). Chemisorption was conducted at two temperatures: 0 °C and 20 °C. A standard gas mixture (10% NH_3_ in He) with the amount of adsorbed gas monitored by the thermal conductivity detector (TCD). Dosing continued until surface saturation was achieved, enabling the calculation of the total adsorbed ammonia. Then, the TPD analysis was performed by heating the sample from the chemisorption temperature up to 250 °C at a rate of 10 °C min^−1^.

## 3. Results

### 3.1. Characterization of Biochars

#### Porous Structure

**Figure 2:**
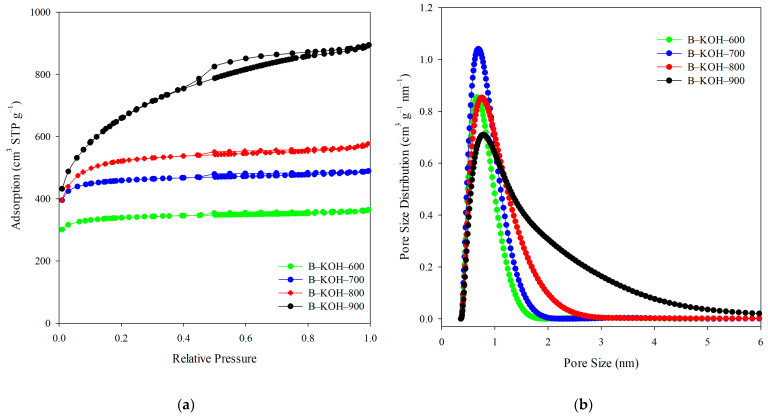
Low temperature N_2_ adsorption/desorption isotherms (**a**) and pore volume distribution curves (**b**) obtained for the biochars.

The nitrogen adsorption-desorption isotherms (Figure 2a) for the three biochars (B–KOH–600, B–KOH–700 and B–KOH–800) exhibit characteristics typical of I type isotherms according to the International Union of Pure and Applied Chemistry (IUPAC) classification [51,52], which indicates the dominant share of micropores in their porous structure. An exception is biochar activated at 900 °C (B–KOH–900), which is IUPAC type IV [51,52], confirming the presence of both micro- and mesopores in the porous structure of the adsorbent. A characteristic hysteresis loop indicates the presence of mesopores of capillary origin, while a steep increase in the volume of N_2_ adsorbed at low relative pressure values indicates well-developed microporosity. The PSD curves (Figure 2b) reveal the presence of a single peak in the micropore range, and their size was estimated by density functional theory (DFT) (Table 1).

The structural parameters calculated based on these isotherms are summarized in Table 1. Non-activated biochar (B) is a material with very low S_BET_ and V_t_ values (S_BET_ = 0.040 m^2^ g^−1^; V_t_ = 0.0014 cm^3^ g^−1^). Due to the low S_BET_ values, nitrogen adsorption/desorption isotherms for this material were not included. The activation of KOH had a very positive effect on the development of the porous structure of the obtained activated biochars. The largest value of S_BET_ was recorded for the B–KOH–900: 2332 m^2^ g^−1^, which places it at the forefront among the analyzed materials. A similar trend applies to the V_t_ (1.38 cm^3^ g^−1^) and the V_micro_ (0.77 cm^3^ g^−1^) of this sample. A reduction in the activation temperature of 800 °C, 700 °C, 600 °C resulted in a smaller extent of porosity development, which is reflected in the lower S_BET_ values (1770 m^2^ g^−1^, 1540 m^2^ g^−1^ and 1134 m^2^ g^−1^, respectively), the total pore (0.89 cm^3^ g^−1^, 0.76 cm^3^ g^−1^ and 0.56 cm^3^ g^−1^) and micropores volumes (0.74 cm^3^ g^−1^, 0.68 cm^3^ g^−1^ and 0.50 cm^3^ g^−1^). Comparing the obtained results, activation at the highest temperature (900 °C) allowed to obtain the specific surface area twice larger than in the case of activation at the lowest temperature (600 °C). These results indicate an evident relationship between the increase in the activation temperature and the increase in the parameters characterizing the porous structure. With the increasing temperature, the S_BET_ and, consequently, the other parameters of the porous structure (V_t_, V_micro_, V_meso_) increase. It should be also noted that in the case of biochar obtained at the highest temperature, not only the volume of micropores but also that of mesopores has been developed. It is worth noting that the micropore sizes decrease with the activation temperature increasing, ranging from 0.78 nm to 0.67 nm (Table 1).

**Figure 3:**
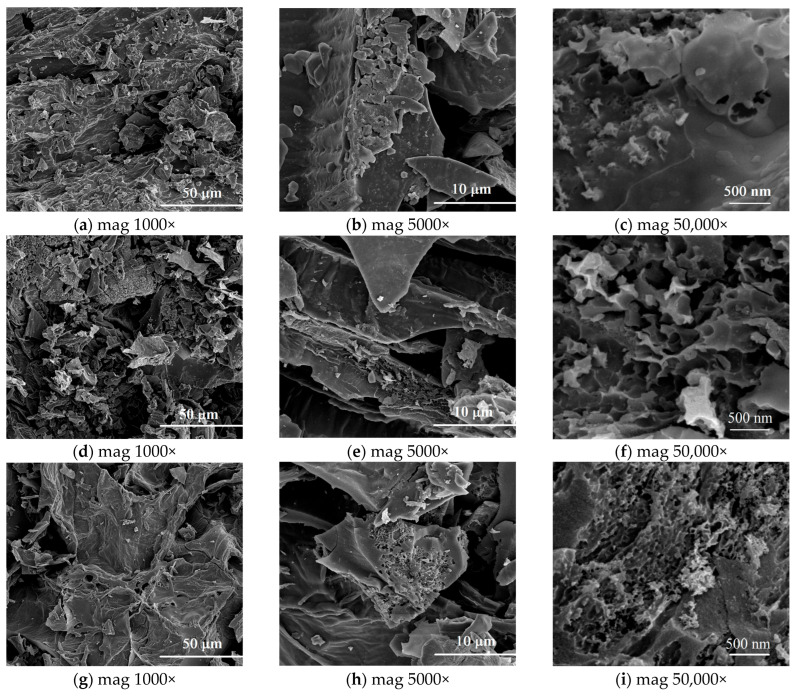

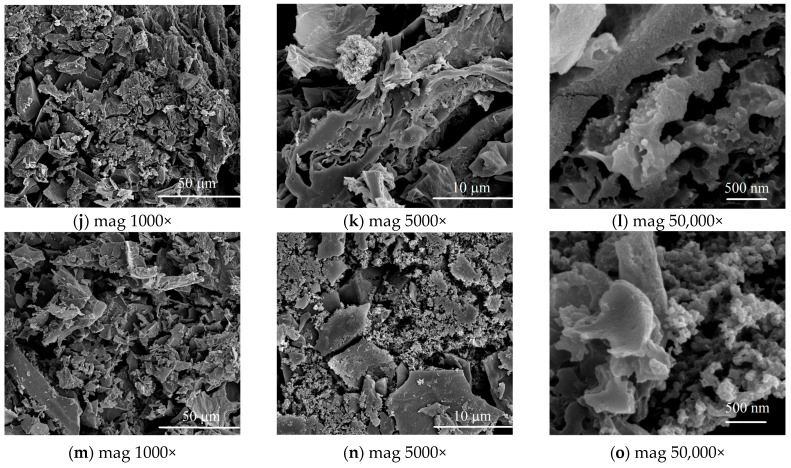
SEM images obtained for non-activated biochar (**a**–**c**) and biochars activated at 600 °C (**d**–**f**), 700 °C (**g**–**i**), 800 °C (**j**–**l**) and 900 °C (**m**–**o**); (mag—magnification).

Scanning electron microscopy (SEM) was used to analyze the surface topography and pore architecture in the obtained biochars. The morphology of biochars depends to a large extent on the properties of the starting material, i.e., banana peels. This factor has a significant impact on the formation of the so-called primary porosity, which is later transformed and developed during the pyrolysis and the activation process. The surface of the non-activated biochar is relatively smooth, compact, and exhibit small porosity (Figure 3 a–c). There are no distinct pores, and the structure does not indicate a developed network of channels, which points out to a limited specific surface area and a small adsorption potential (Table 2). 

**Figure 4:**
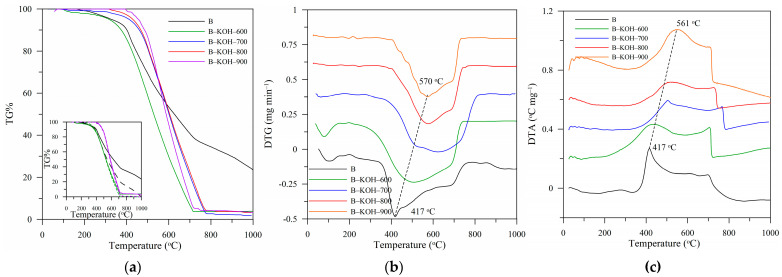
TG% (**a**), DTG (**b**) and DTA (**c**) curves determined for the biochars.

With activation at 600 °C, a porous structure begins to develop (Figure 3 d–f). Single pores of irregular sizes and shapes appear. Activation at this temperature leads to partial surface development but yet it is not fully effective (Table 1). When activated at 700 °C, significant development of porosity is visible (Figure 3 g–i). The structure becomes more open, with clearly defined pores and with greater and better distribution. At magnifications of 5000× and 50,000× there can be observed the formation of channels and cavities, suggesting an effective chemical activation and an increase in the S_BET_ (Table 1). Activation at 800 °C results in further development of the porous structure. The pores are numerous, well-developed, and more uniform in size (Figure 3 j–l). An increase in the pore volume and opening of internal channels are observed, which translates into a significant increase in the surface (Table 1). The highest activated temperature (900 °C) leads to maximum porosity development. The structure of the material is largely deformed, full of branched pores, channels and voids (Figure 3 m–o). However, this can also indicate the beginning of structural degradation at a too high temperature, which can lead to excessive destruction of the pore walls in some cases. This effect of high temperature is also demonstrated by the results of thermal analysis (Figure 4, Table 3) and elemental analysis (Table 4). Nevertheless, porosity and specific surface area reach their maximum here (Table 1).

Figure 4 presents the results of thermal analysis (TG, DTG and DTA) of the biochars. The TG% (Figure 4a) and DTG (Figure 4b) curves indicate that activated biochars have varying thermal stability, especially compared to the initial biochar (B). Activation by KOH caused carbon materials to undergo thermal degradation in a narrower temperature range compared to the starting material (Figure 4c). At the same time, the increase in the temperature of the activation process (from 600 °C to 900 °C) causes the activated biochars to degrade later, which indicates an increase in their thermal stability and better arrangement of graphene structures. This is presented on the TG% curves as a shift in the temperature range of the thermal distribution towards higher values. The TG% curves (Figure 4, inset) show changes in the weight of biochars after correction for moisture and inorganic residue content. The most visible differences in the curves are observed for the initial biochar, due to the weakest arrangement of the carbon material and the largest content of ashes (Table 3). The effectiveness of biochar activation is confirmed by changes in the parameters such as ash content (%A) and volatile (%VC) and solid (%FC, Table 3) carbon content. Activation using KOH at the lowest temperature (B–KOH–600) resulted in a decrease in the ash content (%A) and %VC along with a simultaneous increase in the value of %FC and stability factor (C_thermo_). An increase in the activation temperature intensifies these changes, resulting in a decrease in the ash and %VC content with a simultaneous systematic increase in the %FC content and an increase in the thermostability coefficient C_thermo_ (Table 3).

Table 4 presents a summary of the elemental composition of the obtained biochars. The chemical composition of the analyzed samples shows significant dependence on the temperature used during the activation process. All chemically activated biochars have a large carbon content, ranging from 82.78% to 90.60%. At the same time, small shares of hydrogen (0.288–1.361%), nitrogen (0.21–0.69%) and sulfur (0.127–0.147%) are observed. Contrary to the activated samples, non-activated biochar has a significantly smaller carbon content being 62.43%. Under the conditions of chemical activation of the KOH biochar at the temperatures 600–900 °C, there proceeds a reaction between KOH and mineral components of ash, such as SiO_2_, Al_2_O_3_ or Fe_2_O_3_ which results in formation of soluble silicates, aluminates and other potassium salts, which are then removed during the flushing stage. Therefore, KOH can dissolve partially or transform the ash present in the biochar structure, which affects its final mineral composition and porous structure. The smaller amount of carbon in the non-activated material can be related to the presence of more organic and mineral substances that have not been removed in the activation process. Failure to react with an activating agent (in this case KOH) can result in limited removal of volatile compounds from the carbonaceous material during the high-temperature heating of biomass in N_2_ atmosphere (e.g., gases and organics) and a smaller reduction in the content of undesirable components, leading to an overall reduction in the amount of carbon in the final material. The observed large carbon content in the activated biochars indicates that banana peels can be an effective and valuable waste material for the production of biochars.

**Figure 5:**
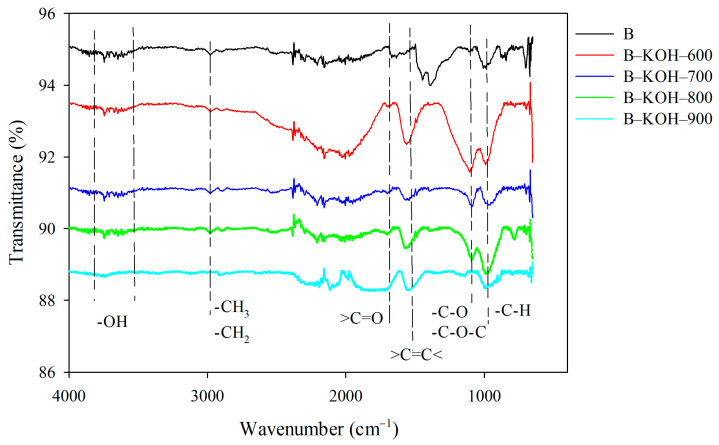
FT-IR spectra of the tested biochars.

The analysis of FTIR spectra (Figure 5) made it possible to identify functional groups present on the surface of the studied biochars. Variation in both the intensity and location of the absorption bands indicates clear differences in chemical structure between the unactivated biochar (B) and the samples after the activation process (B–KOH–600; B–KOH–700; B–KOH–800 and B–KOH–900). The area corresponding to the range of 3800–3300 cm^−1^ can result from the presence of hydroxyl groups (O–H), typical of alcohols, as well as with water molecules adsorbed on the surface [53,54,55,56]. Bands in the range of 3000–2800 cm^−1^ are characteristic for tensile vibrations of C–H bonds in alkyl groups (CH_2_, CH_3_), exactly for the wave number 1892 cm^−1^ with the weakest intensity for B–KOH–900 [57]. On the other hand, signals located between 1700 and 1600 cm^−1^ can be interpreted as a result of the presence of C=C bonds in the aromatic systems or the carbonyl groups (C=O), typical of carboxyl compounds, aldehydes and ketones [58]. The range of 1200–1000 cm^−1^ suggests the presence of C–O bonds, which occur, e.g., in ester structures [57,59]. On the other hand, the region below 1000 cm^−1^ , up to about 400 cm^−1^ , includes bands attributed to deformation vibrations outside the plane of the aromatic rings [55,57]. It should be emphasized that the activation temperature and the activating agent (KOH) have a significant impact on the chemical structure of the surface of the obtained biochars.

**Figure 6:**
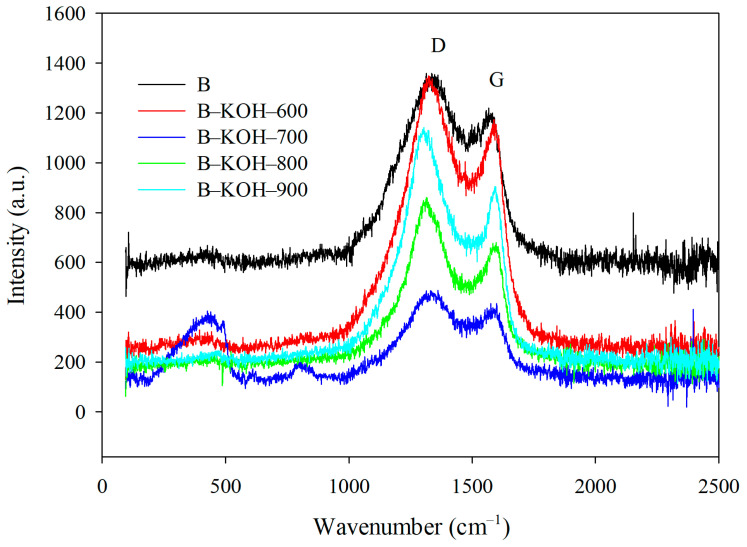
Raman spectra of the tested biochars.

The use of Raman spectroscopy (Figure 6) allowed to estimate the impact of the activation process temperature on the structural arrangement of the analyzed biochars. Two characteristic wide bands typical of carbonaceous materials are observed. They are located near 1350 cm^−1^ and 1600 cm^−1^ , corresponding to the D and G bands. The G-band binds to ordered graphite structures and results from tensile vibrations of carbon-carbon bonds in sp^2^ systems within graphene and graphitized carbon rings. The high intensity and narrow width of this band indicate a high degree of graphitization. In turn, the expansion of the G-band indicates the inherency of defects in the graphene structure. The D-band, absent in the ideal graphite, appears as a result of disturbances in the crystal lattice, signaling the presence of amorphous regions and sp^3^ hybridization structures. Analysis of the intensity ratio of D to G bands (I_D_/I_G_), obtained on the basis of the positions of these bands, allows quantitative assessment of disorder and the degree of crystallinity of samples. An increase in the I_D_/I_G_ value is a clear indicator of an increasing number of defects and less structural order. The values of this ratio (Table 5) in the range of 1.18–1.38, confirm the presence of significant heterogeneities in the carbon structure and a low level of crystallinity of the obtained biochars [60].

The chemical analysis of the surface of biochars, performed by Boehm titration (Table 5), allowed for the determination of the surface acidic and basic functional groups contents. The surface pH of the tested materials was also determined. The obtained values reflect the chemical nature of the surface, which affects directly their adsorption properties. For all the biochars, activated and non-activated ones, both acidic and basic functional groups were identified. Non-activated biochar (B) is clearly alkaline, as evidenced by a large content of basic groups (2.818 mmol g^−1^) and the very high pH of the surface (9.65), Table 5. The content of acidic groups is very small (0.290 mmol g^−1^), which confirms the dominance of basic groups, typical of non-activated carbon materials. This may be due to the lack of action of the activating environment (KOH), which during the activation process at high temperatures promotes the formation of acidic oxygen groups, such as carboxyl, phenolic or lactone ones. Additionally, the non-activated material retains most of the original basic structures, such as pyridine groups, amide groups, or nitrogen atoms containing compounds (if they were present in the precursor). The activation leads to partial degradation of basic groups or their transformation into other surface forms. The introduction of chemical activation using KOH results in significant modification of the surface chemistry of biochar. Already at 600 °C (B–KOH–600), a significant increase in the content of acidic groups (up to 2.300 mmol g^−1^) is observed and a simultaneous decrease in the number of basic groups (up to 0.390 mmol g^−1^), which translates into a significant decrease in the surface pH to 6.68. This change indicates the introduction of oxygen in the form of acidic functionalities into the structure of the material. With the increasing activation temperature (700–900 °C), the content of acidic groups decreases gradually (from 1.978 mmol g^−1^ in B–KOH–700 to 1.248 mmol g^−1^ in B–KOH–900), while the content of basic groups shows an irregular trend. The largest value of basic groups (0.816 mmol g^−1^) was found for biochar activated at 800 °C (B–KOH–800), which may be related to decarboxylation and partial decomposition of acid groups and the appearance of basic surface centers. The total content of functionalities (sum of acidic and basic ones) shows the highest value for the non-activated sample (3.108 mmol g^−1^), but in this case the basic groups predominate. After chemical activation, a more balanced chemical character of the surface is observed. As the activation temperature increases, the surface pH increases from 6.68 (600 °C) to 7.85 (700 °C), after which it decreases slightly to 7.24 (900 °C). This indicates a gradual return to a neutral or slightly alkaline surface character as a result of the decomposition of excess acidic groups at higher temperatures.

The removal efficiency of ammonia (NH_3_) and the results of TPD (thermoprogrammed desorption) analysis of the KOH-activated biochars under different experimental conditions (variable temperatures) are given in Table 2. Compared to non-modified biochar, all activated materials revealed a significantly larger adsorption capacity of NH_3_. It was found that the activation temperature increasing had a significant effect on the lowering of NH_3_ removal efficiency. Moreover, it was shown that the presence of surface groups has a positive impact on the process of ammonia adsorption on the adsorbents surface. Two factors are responsible for the adsorption capacity of the gas phase: porosity and chemical nature of the surface [61,62]. The studied adsorbents are characterized by developed porosity and contain numerous surface functional groups. For the tested materials, the S_BET_ increases with increasing activation temperature (Table 1). However, the highest value of adsorption capacity is obtained for the material with the smallest S_BET_ (B–KOH–600). This suggests that this factor does not play a significant role in the NH_3_ adsorption process. In the adsorption processes of polar adsorbate molecules (NH_3_), the chemical nature of the surface (presence of surface functional groups) plays an important role [33,63]. In the case study, the predominance of acidic groups (Table 5) plays a more important role than porosity (Table 1). The best adsorption properties against ammonia are shown by biochar B–KOH–600 (5.94 mmol g^−1^ (0 °C) and 3.83 mmol g^−1^ (20 °C)), which contains the largest number of acidic functional groups (1.150 mmol g^−1^) among all activated materials and has the lowest S_BET_ (1134 m^2^ g^−1^). The weakest adsorption properties are shown by B–KOH–900 (2.43 mmol g^−1^ (0 °C) and 2.38 mmol g^−1^ (20 °C)), which contains the smallest number of acidic functional groups (0.624 mmol g^−1^) among all activated materials and has the highest specific surface area value (2332 m^2^ g^−1^). The adsorption capacities for B–KOH–700 (4.83 mmol g^−1^ (0 °C) and 3.74 mmol g^−1^ (20 °C)) are smaller than for B–KOH–600, while for B–KOH–800, the values are 3.66 mmol g^−1^ (0 °C) and 3.42 mmol g^−1^ (20 °C), and are lower than for B–KOH–700. In conclusion, as the activation temperature of biochars increases, their adsorption capacity relative to ammonia decreases.

Table 6 compares the NH_3_ adsorption capacities obtained for the tested biochars with other carbon materials discussed in the literature. Analyzing the compiled data, we observe that the materials under investigation are characterized by good adsorption properties against ammonia. To sum up, banana peel waste is a valuable raw material for preparation of activated biochars, while showing a large potential as an effective ammonia (NH_3_) adsorbent in air purification processes.

## 4. Conclusions

This paper presents the preparation and characteristics of biochars from KOH-activated banana peels at temperatures of 600–900 °C. With the increase in the activation temperature, systematic development of the S_BET_ (from 1134 to 2332 m^2^ g^−1^) and porous structure, from the dominance of micropores to the presence of mesopores, was observed. SEM, FTIR, Raman and thermal analyses showed morphological and chemical changes in the materials, including an increase in structural order and modification of functional groups. The largest content of acidic groups (2.30 mmol g^−1^) and the adsorption capacity of NH_3_ (5.94 mmol g^−1^) were obtained for the sample activated at 600 °C. The results indicate that the chemical nature of the surface, rather than the porosity itself, plays a key role in the adsorption of ammonia. It has been shown that the best adsorbent is C–KOH–600, which has the smallest S_BET_ compared to other materials activated at higher temperatures, but it has the largest amount of acidic functional groups. The ammonia molecule is alkaline, so the interaction with the surface on which acidic functional groups are present is clearly facilitated. Therefore, the obtained materials, due to the presence of such functional groups, can be used for adsorption of polar adsorbates of an alkaline nature. Generally, the obtained biochars exhibit large efficiency in NH_3_ removal and competitiveness compared to the materials described in the literature, which confirms the potential of banana peels as a valuable raw material for the production of functional adsorbents.

## Figures and Tables

**Table 1:** Structural parameters for the biochars.

Biochar	S_BET_ (m^2^ g^−1^)	V_t_ (cm^3^ g^−1^)	V_ultra_ (cm^3^ g^−1^)	V_micro_ (cm^3^ g^−1^)	V_meso_ (cm^3^ g^−1^)	w_mi_ (nm)	Micro porosity (%)
B	0.040	0.0014	-	-	-	-	-
B-KOH-600	1134	0.56	0.18	0.50	0.06	0.67	89
B-KOH-700	1540	0.76	0.22	0.68	0.08	0.70	90
B-KOH-800	1770	0.89	0.16	0.74	0.15	0.76	83
B-KOH-900	2332	1.38	0.13	0.77	0.61	0.78	56

**Table 2:** The NH_3_ adsorption capacity and TPD NH_3_ for the studied biochar.

Biochar	NH_3_ Adsorption Capacity (mmol g^−1^) Pressure ~750 mmHg		TPD NH_3_ (mmol g^−1^) Pressure ~750 mmHg	
Temperature	0 °C	20 °C	0 °C	20 °C
B	0.85	0.74	0.25	0.23
B–KOH–600	5.94	3.83	0.71	0.5
B–KOH–700	4.83	3.74	0.65	0.55
B–KOH–800	3.66	3.42	0.59	0.51
B–KOH–900	2.43	2.38	0.45	0.43

**Table 3:** The analysis of ash (%A), carbon content in the form of volatile (%VC) and stable (%FC) structures and thermostability coefficients determined for the studied biochars.

Biochar	%A	%VC	%FC	C_thermo_
B	18.6	33.0	48.4	0.594
B–KOH–600	5.7	26.0	68.3	0.724
B–KOH–700	1.7	12.1	86.2	0.877
B–KOH–800	1.5	8.8	89.7	0.914
B–KOH–900	1.5	8.4	90.0	0.915

**Table 4:** Elemental composition (CHNS) of the obtained biochars.

Biochar	C (%)	H (%)	N (%)	S (%)
B	62.43	1.561	1.26	0.076
B–KOH–600	82.78	1.361	0.69	0.127
B–KOH–700	89.14	0.528	0.19	0.139
B–KOH–800	85.39	0.346	0.22	0.166
B–KOH–900	90.60	0.288	0.21	0.147

**Table 5:** The functional surface groups determined by the Boehm method, the pH and I_D_/I_G_ values of the biochars.

Biochar	Total Basic Groups (mmol g^−1^)	Total Acidic Groups (mmol g^−1^)	Total Groups (mmol g^−1^)	pH_biochar_	I_D_/I_G_
B	2.818	0.290	3.108	9.65	1.18
B-KOH-600	0.390	2.300	2.690	6.68	1.24
B-KOH-700	0.324	1.978	2.302	7.85	1.23
B-KOH-800	0.816	1.416	2.232	7.65	1.38
B-KOH-900	0.590	1.248	1.838	7.24	1.32

**Table 6:** The comparison of the maximum ammonia adsorption capacities of the examined biochars with those reported for various carbon-based adsorbents.

Carbon Materials	Adsorption Capacity (mmol g^−1^)	Ref.
B	0.85	This study
B–KOH–600	5.94	This study
B–KOH–700	4.83	This study
B–KOH–800	3.66	This study
B–KOH–900	2.43	This study
Cu/AC ^1^	7.55	[33]
C–C_1-4_^2^	2.95	[46]
C–C_1-2_^3^	2.61	[46]
C–H_1-4_^4^	2.40	[46]
C–H_1-2_^5^	2.24	[46]
C–O_1-4_^6^	2.78	[46]
C–O_1-2_^7^	2.38	[46]
C–A_1-4_^8^	3.05	[46]
C–A_1-2_^9^	2.59	[46]
OAK–450–KOH ^10^	0.35	[64]
OAK–250–KOH ^11^	1.47	[64]
Na–OH–AC ^12^	1.69	[65]
AC ^13^	1.19	[65]
HNO_3_–AC ^14^	3.07	[65]
AA–WS250–AR ^15^	3.11	[66]
BC–1–CO_2_–6h ^16^	5.18	[59]
BC–2–CO_2_–6h ^17^	3.95	[59]
PP–AC ^18^	4.60	[67]

^1^ mesoporous activated carbon from seaweed waste, activation KOH; ^2−9^ biocarbon from the following trees sawdust: cherry, hornbeam, oak and apple activated KOH; ^10^ biochar from oak wood, activation: KOH; ^11^ hydrochar from oak wood, activation: KOH; ^12^ active carbon impregnation: NaNO_3_ in water; ^13^ active carbon pellets; ^14^ active carbons, activation: HNO_3_; ^15^ biochars from the wood shaving waste, activation: 30% H_3_PO_4_; ^16,17^ biochar from the spruce cones; ^18^ activated carbon from hydrochar of pomelo peel.

## Data Availability

The original contributions presented in this study are included in the article. Further inquiries can be directed to the corresponding author.
